# Knowledge, Awareness, and Attitudes Toward Bone and Soft Tissue Sarcomas Among the General Population in Saudi Arabia: A Cross-Sectional Study

**DOI:** 10.3390/curroncol33040189

**Published:** 2026-03-30

**Authors:** Motaz Alaqeel, Omar A. Aldosari, Abdulrahman Alaseem, Waleed Albishi, Mohammed N. Alhuqbani, Zyad A. Aldosari, Badr Alshehri, Naif Alsaber, Nawaf M. Alwagdani, Ibrahim S. Alshaygy

**Affiliations:** 1 Department of Orthopedic Surgery, College of Medicine, King Saud University, Riyadh 12372, Saudi Arabia; 2 Department of Neurology, Ministry of National Guard—Health Affairs, Riyadh 11426, Saudi Arabia; alsaberna@mngha.med.sa; 3 Department of Orthopedic Surgery, Faisal Specialist Hospital and Research Centre, Riyadh 12713, Saudi Arabia

**Keywords:** bone sarcoma, soft tissue sarcoma, public awareness

## Abstract

**Background:** Bone and soft tissue sarcomas, while rare, making up less than 2% of adult cancers with an incidence below 5 per 100,000 annually, present a significant challenge due to their varied and often obscure pathology. Additionally, the absence of global sarcoma awareness contributes to delayed interventions, necessitating more-aggressive treatments and increasing mortality risks. Conversely, cancers such as breast and colon have seen improved outcomes through effective screening and early-management strategies. **Methods:** In this cross-sectional study, out of the total number of participants approached, using a preset questionnaire, by trained medical students to participate in this study, 626 met the inclusion criteria. The questionnaire started with an informed consent process followed by a set of questions regarding sociodemographic characteristics and lifestyle. Subsequently, the questionnaire delved into their understanding and awareness of bone and soft tissue sarcomas, focusing on risk factors, recognizable signs and symptoms, and tendencies regarding health-seeking behavior. **Results:** In this study with 626 participants, demographic insights showed a young cohort, with 43.5% between 21 and 30 years, and a male predominance of 60.1%. Risk factor awareness was moderate; genetics and smoking were recognized as primary risks for sarcomas. Participants showed limited awareness of sarcoma signs, symptoms, and management, with a substantial percentage unsure about the most at-risk age group, gender differences in risk, and recognizability of symptoms. Barriers to seeking medical care included a passive attitude towards healthcare, fear, and accessibility issues. Most participants had limited knowledge of sarcomas, with 58% unaware of risk factors and 72.3% of signs and symptoms. **Conclusions:** This study emphasizes the necessity for targeted interventions to bridge the knowledge gap and promote early detection practices, which could significantly impact the prognosis of sarcoma patients.

## Simple Summary

Bone and soft tissue sarcomas are rare cancers that develop in bones, muscles, fat, and other body tissues. Because they are uncommon and their early symptoms can resemble harmless conditions, many people are not familiar with them. This lack of awareness may lead to delayed medical consultation and diagnosis, which can result in more-advanced disease and more-complex treatments. In this study, we surveyed 626 members of the public to understand how much people know about these cancers, including their risk factors, warning signs, and when to seek medical care. The results showed that most participants had limited knowledge about sarcomas. Many people were unsure about who is most at risk, what symptoms might suggest a problem, and when medical evaluation is necessary. Some participants also reported barriers to seeking healthcare, including fear, lack of information, and difficulties accessing medical services. These findings highlight an important gap in public knowledge. Increasing public education about bone and soft tissue sarcomas could encourage earlier medical evaluation of suspicious symptoms, leading to earlier diagnosis and better outcomes for patients.

## 1. Introduction

Globally, there has been an increase in the incidence of cancer, which is the leading cause of death worldwide, responsible for nearly 10 million deaths in 2020 [1]. It is anticipated that the number of cancer deaths will continue to rise, reaching 21.4 million worldwide by 2030, as a result of changes in population demographics in the coming decades [2]. Cancer requires a complex and multi-step approach for diagnosis and prognosis. One of the factors is the doctor and patient’s active communication regarding the patient’s awareness of the disease, the diagnostic procedure, the therapeutic options, and the perception of the disease’s prognosis [3]. Another essential factor that determines the clinical outcomes of the patients is health literacy [3].

Sarcomas are a group of heterogeneous cancers that affect soft tissues and bone alike. They are considered rare cancers as their annual incidence is less than 5 per 100.000 people, accounting for <2% of all adult tumors [4]. Bone sarcomas and soft tissue sarcomas include about 100 different pathologic entities as described in the WHO’s Classification of Tumors 2020, many of which are considered ultra-rare, with an incidence of 1 per 1,000,000 people [5]. The obscure nature of bone sarcomas and soft tissue sarcomas and their insidious progression make them prone to late diagnosis. Delays in referral and seeking a second opinion from expert centers all affect the outcomes of sarcoma patients, making them prone to more radical modalities of treatment and increasing the risk of mortality [6]. Furthermore, the number of years of life lost by these patients can be significant because of their young age at presentation [7].

Early diagnosis and treatment of soft tissue and bone sarcomas is crucial for reducing the mortality rate. Individuals with symptoms that may be indicative of sarcoma [8] (e.g., pain, presence of swelling/lump), should be swiftly sent to a sarcoma specialist center, which can perform diagnostic testing, pathology, and therapy as required [6]. This may be achieved by a public health approach aimed at raising sarcoma knowledge, awareness, and health-seeking behaviors [6]. Thus, in this study, we aim to quantify the knowledge and awareness of the general population in Saudi Arabia about bone sarcoma and soft tissue sarcoma, in addition to health-seeking behaviors.

## 2. Methodology

Participants were recruited via convenience sampling from a major high-traffic shopping mall in Riyadh, Saudi Arabia, enabling efficient engagement with a broad segment of the general public during the study period. The study was performed between September 2022 and January 2023. We targeted adults aged 16 years and above who were willing to participate in the study. We excluded patients who were <16 years old and those with cognitive deficits or who could not read or write. The aforementioned individuals were excluded from the study due to their failure to satisfy the research objectives.

The study adhered to the principles of the Helsinki Declaration and received its ethical approval from the Institutional Review Board (IRB) at the College of Medicine, King Saud University, Riyadh, Saudi Arabia (approval no E-22-7328). After being told of the study’s goals and given the option to withdraw at any time without incurring any obligations to the study team, each subject completed a signed consent form indicating their agreement to participate.

To ensure an adequate sample size, reaching at least 420 individuals was the target for enrolment in the study. The calculation of the sample size took into account the current population size of Riyadh, aiming for a 95% confidence level and a margin of error of 5%. We approached more than 1000 individuals: 626 accepted enrollment in the study, and 60 participants were excluded for not meeting the research inclusion criteria or having significant missing data in the research questionnaire.

Questionnaire items were generated following an extensive literature review on cancer awareness, sarcoma epidemiology, and health-seeking behavior surveys. Existing validated awareness instruments were reviewed and selectively adapted to align with the study objectives and local cultural context. Content validity was assessed by a multidisciplinary expert panel comprising orthopedic oncology consultants, epidemiologists, and survey methodology specialists. Panel members evaluated item relevance, clarity, and coverage of key domains, including risk factor awareness, symptom recognition, treatment knowledge, and healthcare access behavior. Recommendations were incorporated iteratively until consensus was achieved on the final instrument structure. To minimize potential self-reported data biases, the study participants were assured that their responses would be kept anonymous and confidential. The questionnaire’s validity was evaluated by survey research experts, and it was designed to minimize fatigue. The questionnaire was divided into five sections. The first section assessed sociodemographic characteristics. The second section had questions regarding desire to change lifestyle. The third section encompassed questions about the knowledge of, awareness of, and attitude toward bone and soft tissue sarcoma. The fourth section dealt with barriers and facilitators to healthcare access. The fifth section comprised items about knowledge-seeking behaviors.

The questionnaire was originally developed in English and subsequently translated into Arabic using a standardized forward–backward translation approach. Forward translation was independently performed by three bilingual translators (two with medical backgrounds and one non-medical professional) to ensure both conceptual accuracy and comprehension. A separate bilingual expert, blinded to the original version, conducted backward translation into English. Discrepancies between versions were reviewed and resolved through consensus discussion among the research team to ensure semantic and conceptual equivalence. The finalized Arabic version was reviewed for cultural appropriateness and readability prior to pilot testing. A pilot study involving 10 participants from the target population was conducted to evaluate the clarity, comprehension, and feasibility of questionnaire completion. Feedback obtained during pilot testing led to minor wording refinements and improved question sequencing. Internal consistency of the knowledge items was assessed using Cronbach’s alpha, demonstrating acceptable reliability (Cronbach’s alpha = 0.643). The data obtained from the pilot sample were not utilized for subsequent analysis. The final version of the questionnaire was distributed in both Arabic and English, and participants were expected to spend approximately 5–6 min to complete it.

Participants were approached at the shopping center by pre-trained medical students and were provided with a consent form explaining the goals of the study and their rights. They were then subsequently given a questionnaire in their preferred language. Medical students were instructed to clarify any questions without providing any hint or clue about the questions. After finishing the questionnaire, the participants were then invited to attend a health awareness campaign about bone and soft tissue sarcoma. Participants were invited to the campaign only after finishing the questionnaire or after refusing to participate in the study. Duplications were minimized by asking if the participant had participated before being invited to participate.

Data was then inserted manually in MS Excel 16.46 (Microsoft, Redmond, WA, USA) and was then cleaned by the removal of any responses with more than 20% missing data per domain. Any missing response of less than 20% per section was assigned as a missing variable in the analysis. Finally, participation with responses that were deemed invalid, such as contradictory responses, was also eliminated.

The research assessed participants’ knowledge and attitudes regarding bone and soft tissue sarcoma. Knowledge assessment was presented using three scores that were calculated based on the participants’ responses. The first score was about the knowledge of risk factors for bone and soft tissue sarcoma. This score was calculated by adding the number of correct responses for questions 11 to 13. The second score measured the knowledge of signs and symptoms of bone and soft tissue sarcoma. This measure rose by adding the number of correct responses for question 14. The third measure regarded the knowledge of management of bone and soft tissue sarcoma, which corresponded to the number of correct responses for question 16. The attitude assessment was assessed using questions 15, 21, 22, and 23.

Qualitative variables are presented using numbers and percentages in the form of descriptive statistics, while the mean and standard deviation are utilized for quantitative variables. To examine the association of the total barriers and facilitator scores with sociodemographic characteristics, the Mann–Whitney Z-test and Kruskal–Wallis H-test were employed. Associations were examined using a univariable Logistic regression model, reporting odds ratios (ORs) and 95% confidence intervals (CIs). Data analysis was conducted using IBM Statistical Packages for Social Sciences (SPSS) Statistics for Windows, version 26 (IBM Corp., Armonk, NY, USA). Statistical significance was determined at a threshold of *p* < 0.05.

## 3. Results

**Figure 1:**
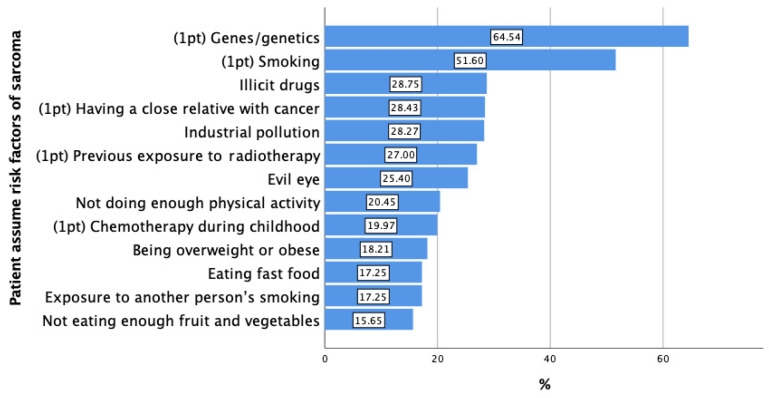
General population assumed risk factors of soft tissue and bone sarcomas.

This study of 626 participants showed varied age demographics, with 43.5% aged 21–30 and a 60.1% male majority. Education-wise, 45.8% had a bachelor’s degree, and employment figures showed that 55.6% were working, with 23.2% unemployed. Income levels varied, with 17.4% earning under 5000 SAR. Notably, 64.9% had no family cancer history, while 32.1% were smokers. Interest in lifestyle changes varied, with only 7.0% strongly inclined to change. These demographics provide insights into the educational, economic, and health characteristics of the cohort (Table 1). The population study indicates that 64.54% consider genetics a primary sarcoma risk, with 51.60% identifying smoking. Other risks include illicit drugs at 28.75%, a cancerous family history at 28.43%, and exposure to industrial pollutants at 28.27%. Radiotherapy exposure concerns 27.00%, and the evil eye is believed to be a factor by 25.40%. Lesser risks include insufficient exercise at 20.45%, childhood chemotherapy at 19.97%, and obesity at 18.21%. Fast-food consumption and second-hand smoke are noted by 17.25%, and a fruit- and vegetable-deficient diet is a concern for 15.65% (Figure 1).

Participants’ knowledge concerning soft tissue and bone sarcomas reveals that when asked about the age group most at risk for developing sarcomas, 22.7% believed it to be between 31 and 50 years, while 29.6% were unsure. Regarding gender susceptibility, 39.8% perceived no difference in sarcoma risk between males and females, and 39.9% were uncertain. In identifying warning signs of bone and soft tissue sarcomas, the most recognized symptom was an unexplained lump or swelling at 32.6%. Persistent unexplained pain was acknowledged by 33.5%, and 33.7% were unsure about the signs altogether. Regarding bone sarcoma treatability, 49.7% responded affirmatively, while 43.9% expressed uncertainty. Finally, when asked about management modalities for bone and soft tissue sarcomas, radiotherapy was identified by 35.6% of participants and limb-salvaging surgery was mentioned by 22.2%, while 43.8% acknowledged a lack of knowledge on the subject (Table 2).

**Figure 2:**
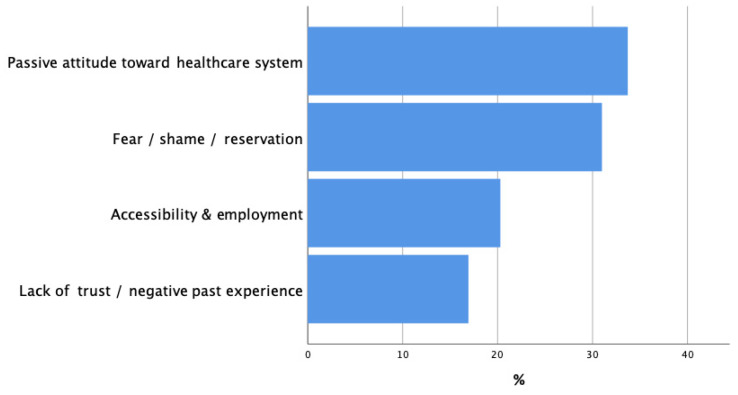
Barriers to seeking medical attention.

**Figure 3:**
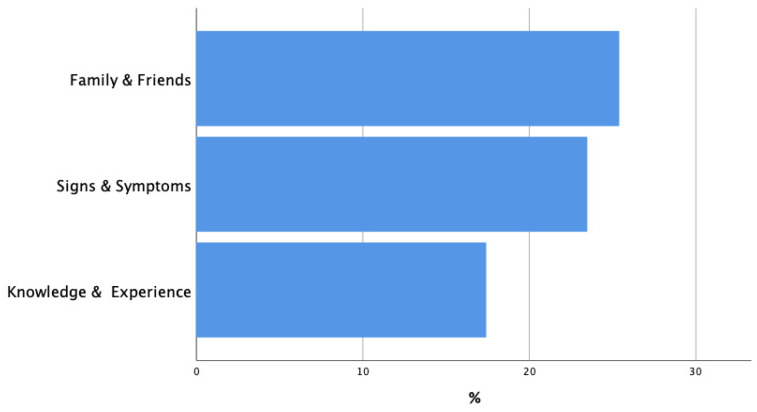
Facilitators of seeking medical attention.

The primary barriers influencing individuals’ decisions to seek medical attention include a passive attitude toward the healthcare system at approximately 40%; feelings of fear, shame, or reservation at around 30%; concerns related to accessibility and employment at about 25%; and negative past experiences or lack of trust in the healthcare system at close to 10%. In contrast, the main facilitators driving individuals to seek medical care are encouragement from family and friends at roughly 30%, recognizing specific signs and symptoms at about 25%, and one’s knowledge and past experiences with the medical system at approximately 10% (Figure 2 and Figure 3).

The study found that most participants had limited knowledge about sarcoma, with 58% showing poor awareness of risk factors and 72.3% having a poor understanding of signs and symptoms. Only 7% and 3.7% had good knowledge in these areas, respectively. Additionally, 57.5% had poor knowledge of sarcoma management. A majority, 70.1%, believed cancer prevention is possible, while 29.9% disagreed. Information-seeking was observed in 40.9% of the sample, with 51.6% relying on non-official sources. Social media was considered a trustworthy source by 59.9%. Opinions on responsibility for early cancer detection varied: 42.5% viewed it as a shared responsibility, 28.3% felt it was an individual’s duty, 9.6% assigned it to the healthcare system, and 19.7% were uncertain (Table 3).

In the survey, 40.9% of participants actively sought cancer information, while 40.1% did not, and 18.8% could not recall. Family and friends were the primary sources for 35%, followed by official medical websites at 30.8%, and interactions with cancer patients or survivors at 21.4%. The Saudi Orthopedics Association website was used by 11.7%, healthcare providers by 16.9%, and non-medical websites by 16.1%, and a notable 41.7% turned to social media. Other sources were cited by 16.9%. For social media, 23.5% preferred Twitter and Facebook, 15.5% YouTube, and 12.9% various platforms. Snapchat/TikTok and Instagram were less trusted, at 4.6% and 1.6%, with 41.9% distrusting social media for cancer information (Table 4).

In a sarcoma awareness study with 626 participants, age emerged as a significant factor; those over 30 had a better grasp of sarcoma risks (OR = 2.10, 95% CI [1.6–2.7], *p* < 0.001) and management (OR = 0.44, 95% CI [0.27–0.72], *p* = 0.001) than their younger counterparts. Women surpassed men in recognizing symptoms (OR = 1.29, 95% CI [0.92–1.80], *p* = 0.030). Higher education, specifically a bachelor’s degree or more, was linked to better management knowledge (OR = 1.66, 95% CI [1.07–2.57], *p* = 0.022). Income levels above 10,000 SAR were associated with greater awareness of risk factors (OR = 1.75, 95% CI [1.29–2.37], *p* = 0.024) and management (OR = 0.39, 95% CI [0.24–0.64], *p* = 0.022). Employment positively affected understanding of risks (OR = 0.50, 95% CI [0.31–0.80], *p* = 0.033) and management (OR = 0.49, 95% CI [0.31–0.79], *p* = 0.022). The desire for lifestyle changes was significant, as those with moderate to solid intention had better overall knowledge, notably in risks (OR = 0.32, 95% CI [0.1–0.8], *p* = 0.011) and management (OR = 0.26, 95% CI [0.1–0.67], *p* = 0.001) (Table 5).

In the analysis of demographic factors and their association with attitudes towards sarcomas, younger participants (<30 years) tended to use non-official sources for cancer information (OR = 0.46, 95% CI [0.29–0.74], *p* = 0.021) and trust social media for such data (OR = 0.61, 95% CI [0.41–0.90], *p* = 0.011). Higher education correlated with less reliance on non-official sources (OR = 0.48, 95% CI [0.31–0.74], *p* = 0.001). Those earning over 10,000 SAR monthly were more proactive regarding early cancer detection (OR = 0.29, 95% CI [0.14–0.59], *p* = 0.001). A family cancer history significantly increased the pursuit of cancer information (OR = 1.8, 95% CI [1.3–2.5], *p* < 0.001) and the belief in healthcare’s role in detection (OR = 0.47, 95% CI [0.25–0.87], *p* = 0.015). Individuals desiring lifestyle changes were more likely to trust social media (OR = 0.46, 95% CI [0.3–0.70], *p* = 0.003) and to accept personal responsibility in detecting cancer early (OR = 0.48, 95% CI [0.28–0.82], *p* = 0.007) (Table 6).

## 4. Discussion

The management and prognosis of any form of cancer can be aided by knowledge and awareness of the risk factors and early symptoms of the disease. Bone and soft tissue sarcomas often do not initially exhibit any signs that would allow for an early diagnosis. In addition, young people and adolescents are more likely than older adults and children to develop bone and soft tissue sarcomas; thus, despite lower incidence rates, the number of years of life lost may frequently be significant [9]. Therefore, raising awareness of the disease’s risk factors and early warning signs can help with disease prevention and early identification, which can enhance the prognosis overall [10]. This study, which sought to assess general Saudi Arabians’ knowledge and awareness of various aspects of bone and soft tissue sarcomas, was conducted in light of the paucity of research on sarcomas and knowledge and awareness of them among the general population.

We have examined the general Saudi population’s knowledge and attitudes on bone and soft tissue sarcomas in light of several demographic parameters. The results of our study show that, in comparison to males, women exhibit better levels of awareness about the risk factors, early symptoms, and markers of malignancies. This result is consistent with earlier studies that have repeatedly shown that women are more knowledgeable about different tumors. Societal, cultural, and biological variables may all play a role in this disparity [11,12,13]. Women usually prioritize their health and interact more regularly with healthcare institutions due to societal expectations and gender roles, which increases awareness of diseases. In addition, biological aspects like women’s greater breast cancer prevalence and the emphasis on breast health awareness campaigns may help women have a deeper understanding of tumors in general [14,15].

Additionally, the findings of this study indicate a positive correlation between higher education levels and knowledge concerning the signs and symptoms of bone and soft tissue sarcomas. Similarly, individuals with higher educational attainment and higher family income demonstrated greater awareness regarding the management strategies for bone and soft tissue sarcomas. These results suggest that socioeconomic factors, such as education level and income, play a significant role in shaping the level of knowledge within the community regarding these malignancies. This is consistent with earlier research that looked at the relationship between educational attainment and financial stability and knowledge of various cancer types [11,13].

Furthermore, our study investigated the association between smoking practices and awareness of bone and soft tissue sarcomas. Our findings show that smokers had lower knowledge and awareness of sarcoma risk factors, symptoms, and warning signs compared to non-smokers. This may reflect less engagement with preventive health behaviors and limited exposure to formal health information. Sociodemographic factors, such as education, occupation, and health literacy, may also influence this relationship. Interpret this association cautiously, as the cross-sectional study design precludes causal inference regarding the relationship between smoking status and knowledge or awareness. Residual confounding may exist, and the relationship may reflect broader behavioral or socioeconomic factors rather than smoking status alone. Nevertheless, these findings underscore the value of targeted awareness interventions for high-risk behavioral groups to improve early recognition and health-seeking behavior. This finding is consistent with other research, which has consistently demonstrated that smokers frequently lack basic understanding of malignancies associated with smoking [16].

In light of the potential consequences for early identification, prevention, and treatment outcomes, it is important to note that addressing this knowledge gap among smokers is crucial. In order to provide people with the resources they need to make wise health decisions, efforts should be made to increase awareness and offer tailored educational interventions that are explicitly directed towards smokers.

We additionally investigated the awareness of bone and soft tissue sarcomas and whether or not there was a family history of cancer. It turned out that there was no difference in knowledge of the risk factors and the signs and symptoms of these cancers between people with and without a family history of cancer, which is intriguing, as it goes against earlier research that found that people with a family history of cancer frequently have better knowledge of the risk factors [17].

Nonetheless, according to our study, individuals with a history of cancer in their families were more knowledgeable about the management of bone and soft tissue sarcomas than people without a family history.

This result raises the likelihood that those who have seen or experienced cancer in their relatives may be more motivated to research and educate themselves about the illness. Through a family member’s experience, people may become more aware of cancer and be motivated to actively learn about the curative regimens and assistance programs available for bone and soft tissue sarcomas. This highlights the importance of considering the impact of family history when developing educational initiatives and support programs. Tailoring interventions to address the specific needs of individuals with a family history of cancer can empower them with the knowledge required to make informed decisions, actively engage in their healthcare, and potentially contribute to better outcomes in the management of bone and soft tissue sarcomas.

We also looked at the connection between people’s motivation to alter their lifestyle and their familiarity with various aspects of bone and soft tissue sarcomas. Surprisingly, our research found a link between people’s understanding of the risk factors, symptoms, and treatment options for these malignancies and how strongly they desired to change their lifestyles.

According to this finding, those who show a mild, moderate, or strong desire to make changes to their lifestyles have, on average, more information about bone and soft tissue sarcomas than people who have a weak desire to do so. It is plausible that people who are driven to make better choices are more likely to get informed about the risks associated with those choices.

This finding highlights the significance of promoting a desire for lifestyle changes in order to boost cancer awareness and knowledge. A more informed populace can be achieved by motivating people to actively make healthier decisions and by giving them access to relevant knowledge and resources. We can promote early detection, prevention, and generally improved outcomes in the field of bone and soft tissue sarcomas by educating people about risk factors, signs, symptoms, and management techniques.

A prerequisite for the encouragement of preventive or screening practices is the healthcare provider’s belief that a condition or its worst prognostication may be avertible when detected early in its natural course; thus, we aimed to assess whether our participants shared the ascertainment that sarcomas are preventable. Our results show that 70.1% of our respondents believe that sarcomas can be prevented. This is higher than what was reported by the 1987 Health Information Survey, in which 44% of the participants believed that there was nothing they could do to alter their risk of acquiring cancer [18]. Similarly, a study among Arab women in Qatar showed that 57.2% of the respondents believed in the nonpreventability of cancer [19]. The reasons for such disparity are subject to speculation; however, the nonuniformity in the choices presented may have contributed to the difference in the results, as our questionnaire allowed for an equivocal answer, whereas the former study adopted a dichotomous approach in answering this question.

Moreover, information-seeking behavior was reported only by 40.9% of our respondents. The arbitrary definition of information-seeking may have conceivably confounded the result; as such, these questions are probably best answered through a qualitative approach or by careful delineation of what constitutes an information-seeking behavior. The majority of our respondents reported the use of non-official sources in finding cancer-related information; expectedly, this was also reflected by the trust of 59.9% of our participants in social media as a source of information. Social media platforms are channels through which both official and non-official sources can conveniently be navigated. Moreover, the use of social media may persist in spite of low self-reported trust [20]. The majority of our participants believe early cancer detection to be a responsibility that at least in part involves personal agency, with 42.5% proclaiming it to be a shared responsibility between the individual and the healthcare provider, and 28.3% believing it to be solely a matter of personal responsibility. Overall, these findings reassuringly indicate a low prevalence of medical nihilism among our participants; this, coupled with the belief in personal agency in cancer management, suggests fertile grounds to sow the seeds of health-promoting behavior.

We are aware of the limitations of our study; the closed-ended approach precluded the exploration of more personal viewpoints unaddressed by the self-administered questionnaire. Furthermore, recruiting from a single shopping mall through convenience sampling may compromise the representativeness of the study population, as mall-based surveys may differentially capture specific demographic and socioeconomic groups. Additionally, the study was geographically limited to Riyadh, Saudi Arabia; thus, the generalizability of our findings cannot be assumed for the entire region, and they are evidently in need of replication. 

This study underscores the substantial gap in public awareness and understanding of bone and soft tissue sarcomas. The demographic profile of the participants, predominantly young adults with a male majority, reflects a segment of the population that is relatively unaware of the risk factors, signs, and symptoms associated with these sarcomas. In light of these findings, there is a critical need for targeted educational initiatives and awareness campaigns that focus on the unique aspects of bone and soft tissue sarcomas. Such efforts should aim to improve the general public’s understanding of these cancers, including their risk factors, symptoms, and available treatments. Emphasis should also be placed on encouraging proactive health-seeking behaviors and guiding individuals towards credible sources of medical information. By bridging this knowledge gap, we can potentially expedite early detection and intervention, thereby improving the prognosis and outcomes for those affected by these challenging cancers.

## Figures and Tables

**Table 1:** Sociodemographic and lifestyle characteristics of participants (n = 626).

Study Data	N (%)
Age group	
≤20 years	126 (20.1%)
21–30 years	272 (43.5%)
31–40 years	135 (21.6%)
>40 years	93 (14.9%)
Gender	
Male	376 (60.1%)
Female	250 (39.9%)
Educational level	
High school or below	156 (24.9%)
Diploma	79 (12.6%)
Bachelor’s degree	287 (45.8%)
Higher degree [master’s degree & PhD]	104 (16.6%)
Family monthly income (SAR) ^(n = 594)^	
<5000	109 (17.4%)
5000–9999	193 (30.8%)
10,000–20,000	158 (24.3%)
>20,000	136 (21.7%)
Employment status	
Unemployed	145 (23.2%)
Employed	348 (55.6%)
Student	132 (21.1%)
Family history of cancer	
No family history	406 (64.9%)
Yes, first-degree relative	98 (15.7%)
Yes, second-degree relative	122 (19.5%)
Smoking Status	
Smoker [current, past, E-cig]	201 (32.1%)
Non-smoker	425 (67.9%)
Desire to change lifestyle (score)	
Poor (0)	20 (3.2%)
Mild (1–2)	382 (61.0%)
Moderate (3–4)	180 (28.8%)
Strong (>4)	44 (7.0%)

**Table 2:** Soft tissue and bone sarcoma knowledge (n = 626).

Knowledge Statement	N (%)
At what age would someone be at most risk of developing sarcomas?	
0–30 years	58 (9.3%)
31–50 years	142 (22.7%)
>50 years	76 (12.1%)
It is not related to age	122 (19.5%)
It depends on the sarcoma type [1 pt]	43 (6.9%)
I don’t know	185 (29.6%)
Which gender is more likely to develop sarcoma?	
Male	62 (9.9%)
Female	62 (9.9%)
No difference [1pt]	249 (39.8%)
I don’t know	250 (39.9%)
Which of the following are warning signs of bone & soft tissue sarcomas?	
An unexplained lump or swelling [2pt]	204 (32.6%)
Unexplained weight loss [1pt]	138 (22.0%)
Fracture from small fall or accident [1pt]	106 (16.9%)
Unexplained bleeding	72 (11.5%)
Persistent unexplained pain [1pt]	210 (33.5%)
Unexplained limp [1pt]	88 (14.1%)
Fatigue and tiredness	119 (19.0%)
Fever	55 (8.8%)
I don’t know	211 (33.7%)
Do you think bone sarcomas can be treated?	
Yes [1pt]	311 (49.7%)
No	39 (6.2%)
I don’t know	275 (43.9%)
Which of the following can be considered in the management of bone & soft tissue sarcomas?	
limb salvaging surgery (limb sparing) [0.25 pt]	139 (22.2%)
Amputation of affected limb [0.25 pt]	73 (11.7%)
Chemotherapy [0.25 pt]	223 (35.6%)
Radiotherapy [0.25 pt]	115 (18.4%)
I don’t know	274 (43.8%)

**Table 3:** Knowledge and attitudes of participants (n = 626).

Knowledge and Attitude (Scores)	N (%)
Knowledge:	
Risk factors	
Poor (0–2)	363 (58.0%)
Moderate (3–4)	219 (35.0%)
Good (>4)	44 (7.0%)
Signs & Symptoms	
Poor (0–2)	455 (72.3%)
Moderate (3–4)	219 (35.0%)
Good (>4)	23 (3.7%)
Management	
Poor (0–1)	360 (57.5%)
Moderate (2–3)	230 (36.7%)
Good (>3)	36 (5.8%)
Attitude:	
Belief that cancer can be prevented	
Yes	444 (70.1%)
No	181 (29.9%)
Previous cancer information-seeking:	
Yes	256 (40.9%)
No	369 (18.8%)
Information-seeking behavior:	
Official sources	92 (14.7%)
Non-official sources	323 (51.6%)
Both	191 (30.5%)
Neither	20 (3.2%)
Trust in social media as a source of cancer information:	
Yes	370 (59.9%)
No	256 (40.1%)
Responsibility attitude towards early cancer detection	
Individuals’ responsibility	177 (28.3%)
Healthcare system responsibility	60 (9.6%)
A shared responsibility	266 (42.5%)
Not sure	123 (19.7%)

**Table 4:** Sarcomas and cancer information-seeking behavior (n = 626).

Study Data	N (%)
Have you ever looked for information about cancer from any source?	
Yes	256 (40.9%)
No	251 (40.1%)
Don’t remember	118 (18.8%)
What sources of information have you used to find out about cancer?	
Friends or family	219 (35.0%)
Official medical websites	193 (30.8%)
Cancer patient/survivor	134 (21.4%)
Saudi Orthopedics Association website	73 (11.7%)
Healthcare provider	106 (16.9%)
Non-medical websites	101 (16.1%)
Social media	261 (41.7%)
Other	106 (16.9%)
Which of the following social media platforms you trust regarding medical information and cancer?	
Twitter/Facebook	147 (23.5%)
Snapchat/TikTok	29 (4.6%)
Instagram	10 (1.6%)
YouTube	97 (15.5%)
Multiple platforms	81 (12.9)
I don’t trust social media	262 (41.9%)

**Table 5:** Association between sarcoma knowledge and demographic factors (n = 626).

	Knowledge											
	Risk Factors				Signs & Symptoms				Management			
Factor	Poor	Moderate	Good	*p*	Poor	Moderate	Good	*p*	Poor	Moderate	Good	*p*
Age												
≤20 years	88	35	1	0.1	93	27	4	0.9	78	36	10	0.3
21–30 years	176	81	16		194	67	12		156	103	14	
≥30 years	141	76	9		166	53	7		125	89	12	
Gender												
Male	277	88	11	<0.000	290	73	13	0.007	228	127	21	0.14
Female	128	107	15		165	75	10		132	103	15	
Educational level												
Diploma or below	164	65	6	0.071	188	42	5	0.005	147	81	7	0.027
Bachelor’s or higher	241	130	20		106	18	106		213	149	29	
Family monthly income (SAR)												
<10,000	190	94	18	0.094	224	67	11	0.723	168	123	11	0.024
≥10,000	188	97	7		208	72	12		173	96	23	
Employment status												
Unemployed	174	112	8	0.001	211	74	9	0.561	165	111	18	0.806
Employed	230	83	18		243	74	14		194	119	18	
Smoking status												
Smoker	123	30	6	<0.000	127	26	6	0.043	98	56	5	0.194
Non-smoker	282	165	20		328	122	17		262	174	31	
Family history of cancer												
No	306	86	14	0.119	240	150	16	0.029	123	30	6	<0.000
Yes	149	62	9		120	80	20		282	165	20	
Desire to change lifestyle												
Poor	14	5	1	<0.000	14	3	3	<0.000	13	7	0	<0.000
Mild	278	96	8		310	62	10		236	137	9	
Moderate	90	79	11		101	69	10		89	69	22	
Strong	23	15	6		30	14	0		22	17	5	

**Table 6:** Association between sarcoma knowledge and participants’ attitudes (n = 626).

	Knowledge											
	Risk Factors				Signs & Symptoms				Management			
Attitude	Poor	Moderate	Good	*p*	Poor	Moderate	Good	*p*	Poor	Moderate	Good	*p*
Belief that cancer can be prevented												
Yes	268	154	22	0.006	307	117	20	0.030	241	173	30	0.151
No	35	6	0		38	3	0		26	15	0	
Not sure	102	34	4		109	28	3		92	42	6	
Seeking information about cancer												
Yes	91	59	10	0.037	107	40	13	0.002	73	69	18	0.000
No	314	136	16		348	108	10		287	161	18	
Information-seeking behavior:												
Official sources	66	22	4	0.001	68	21	3	0.000	52	34	6	0.000
Non-official sources	227	86	10		265	54	4		212	104	7	
Both	112	87	12		122	73	16		96	92	23	
Trust social media as a source of cancer information:												
Yes	219	127	18	0.019	262	82	20	0.015	203	141	20	0.473
No	186	68	8		193	66	3		157	89	16	
Responsibility attitude towards early cancer detection												
Individuals’ responsibility	116	57	4	0.007	142	33	2	0.001	97	72	8	0.001
Healthcare system responsibility	44	15	1		46	13	1		38	21	1	
A shared responsibility	151	97	18		168	81	17		137	104	25	
Not sure	94	26	3		99	21	3		84	44	2	

## Data Availability

The datasets used in this study are available from the corresponding author on reasonable request.
